# Supplementary material for: Anticipatory behaviour as an indicator of the welfare of dairy calves in different housing environments
Source: PLoS One. 2021 Jan 20;16(1):e0245742. doi: 10.1371/journal.pone.0245742 (PMC7816972; doi:10.1371/journal.pone.0245742)
Supplement: S1 Table — Following a 1 min anticipatory period in the home pen, behaviours expressed during the 30 min access period in the reward pen (basic or enriched, n = 3 each). Behaviours are presented as percentage of total duration (± SD) in the reward pen for the test phase (average of day 13 to 15), for the day after a change in reward pen quality1 (day 17), and for subsequent days of the contrast phase (average of day 18 to 20). Data is presented for descriptive purposes only due to data loss. (DOCX) [file pone.0245742.s001.docx]

**S1 Table. Behaviours expressed in the reward pen.**

| **Reward pen treatment** | **Walking (% of period)** | **Standing inactive (% of period)** | **Exploring (% of period) ^2^** | **Play  (% of period) ^3^** | **Grooming (% of period)** | **Rope use (% of period) ^4^** | **Brush use  (% of period) ^4^** |
| --- | --- | --- | --- | --- | --- | --- | --- |
| Test phase |  |  |  |  |  |  |  |
| Basic | 7.2 ± 3.1 | 16.6 ± 6.8 | 55.7 ± 17.3 | 12.7 ± 5.7 | 4.6 ± 1.7 | N/A | N/A |
| Enriched | 5.1 ± 0.7 | 18.9 ± 4.4 | 39.4 ± 6.6 | 9.3 ± 2.9 | 4.4 ± 0.9 | 7.4 ± 2.5 | 10.8 ± 8.3 |
| Contrast phase |  |  |  |  |  |  |  |
| Changed to Basic |  |  |  |  |  |  |  |
| Day 17 | 5.3 ± 2.1 | 17.1 ± 5.9 | 66.0 ± 6.7 | 4.3 ± 2.2 | 5.4 ± 4.6 | N/A | N/A |
| Day 18-20 | 5.4 ± 0.9 | 16.8 ± 4.3 | 56.2 ± 17.5 | 5.9 ± 4.7 | 2.5 ± 4.0 | N/A | N/A |
| Changed to Enriched |  |  |  |  |  |  |  |
| Day 17 | 5.4 ± 0.5 | 13.0 ± 1.7 | 37.7 ± 3.6 | 9.8 ± 5.6 | 2.2 ± 2.3 | 11.2 ± 5.9 | 20.0 ± 15.9 |
| Day 18-20 | 6.4 ± 0.04 | 16.9 ± 0.3 | 43.6 ± 15.2 | 11.2 ± 2.1 | 2.5 ± 1.2 | 6.9 ± 5.7 | 11.6 ± 11.2 |

Behaviours expressed during the 30 min access period (following a 1 min anticipatory period in the home pen) in the reward pen (basic or enriched, n = 3 each). Behaviours are presented as percentage of total duration (± SD) in the reward pen for the test phase (average of day 13 to 15), for the day after a change in reward pen quality^1^ (day 17), and for subsequent days of the contrast phase (average of day 18 to 20). Data is presented for descriptive purposes only.

^1^ Reward pen treatment was changed for the contrast phase (from basic to enriched and vice versa, for all calves). Before the change (day 13 to 15), calves entered the reward pen expecting the original reward pen. On day 16, the reward pen was changed (transition day, data not presented). Calves entered the reward pen expecting the new reward pen on the first day after the change (day 17), and subsequent days of the contrast phase (day 18 to 20).

^2^ Sum of the percentage of duration of touching wall and floor + licking wall + licking floor (stones in basic pen)

^3^ Sum of the percentage of duration of locomotory play + social play

^4^ Not applicable for basic reward pen (did not contain rope or brush)
