# Supplementary material for: Anticipatory behaviour as an indicator of the welfare of dairy calves in different housing environments
Source: PLoS One. 2021 Jan 20;16(1):e0245742. doi: 10.1371/journal.pone.0245742 (PMC7816972; doi:10.1371/journal.pone.0245742)
Supplement: Attachment — Submitted filename: Response to Reviewers - Revision 1.docx [file pone.0245742.s002.docx]

**Response to Reviewers – PloS ONE. Anticipatory behaviour as an indicator of the welfare of dairy calves in different housing environments**

Journal Requirements:

When submitting your revision, we need you to address these additional requirements.

1. Please ensure that your manuscript meets PLOS ONE's style requirements, including those for file naming. The PLOS ONE style templates can be found at

https://journals.plos.org/plosone/s/file?id=wjVg/PLOSOne_formatting_sample_main_body.pdf and

<https://journals.plos.org/plosone/s/file?id=ba62/PLOSOne_formatting_sample_title_authors_affiliations.pdf>

AU: Corrected

2. Please include captions for your Supporting Information files at the end of your manuscript, and update any in-text citations to match accordingly. Please see our Supporting Information guidelines for more information: [http://journals.plos.org/plosone/s/supporting-information](about:blank).

AU: Corrected

3.Thank you for stating the following in the Financial Disclosure section:

[The study funding was provided by AgResearch Core funding (A19041). HN was supported by the Natural Science and Engineering Research Council Canada Graduate Scholarship and the Michael Smith Foreign Study Supplement.].

We note that one or more of the authors have an affiliation to the commercial funders of this research study : AgResearch Ltd.

1. Please provide an amended Funding Statement declaring this commercial affiliation, as well as a statement regarding the Role of Funders in your study. If the funding organization did not play a role in the study design, data collection and analysis, decision to publish, or preparation of the manuscript and only provided financial support in the form of authors' salaries and/or research materials, please review your statements relating to the author contributions, and ensure you have specifically and accurately indicated the role(s) that these authors had in your study. You can update author roles in the Author Contributions section of the online submission form.

Please also include the following statement within your amended Funding Statement.

“The funder provided support in the form of salaries for authors [insert relevant initials], but did not have any additional role in the study design, data collection and analysis, decision to publish, or preparation of the manuscript. The specific roles of these authors are articulated in the ‘author contributions’ section.”

If your commercial affiliation did play a role in your study, please state and explain this role within your updated Funding Statement.

AU: Corrected and added to coverletter as requested.

2. Please also provide an updated Competing Interests Statement declaring this commercial affiliation along with any other relevant declarations relating to employment, consultancy, patents, products in development, or marketed products, etc.

Within your Competing Interests Statement, please confirm that this commercial affiliation does not alter your adherence to all PLOS ONE policies on sharing data and materials by including the following statement: "This does not alter our adherence to  PLOS ONE policies on sharing data and materials.” (as detailed online in our guide for authors [http://journals.plos.org/plosone/s/competing-interests](about:blank)). If this adherence statement is not accurate and  there are restrictions on sharing of data and/or materials, please state these. Please note that we cannot proceed with consideration of your article until this information has been declared.

Please include both an updated Funding Statement and Competing Interests Statement in your cover letter. We will change the online submission form on your behalf.

Please know it is PLOS ONE policy for corresponding authors to declare, on behalf of all authors, all potential competing interests for the purposes of transparency. PLOS defines a competing interest as anything that interferes with, or could reasonably be perceived as interfering with, the full and objective presentation, peer review, editorial decision-making, or publication of research or non-research articles submitted to one of the journals. Competing interests can be financial or non-financial, professional, or personal. Competing interests can arise in relationship to an organization or another person. Please follow this link to our website for more details on competing interests: [http://journals.plos.org/plosone/s/competing-interests](about:blank)

AU: Corrected and added to coverletter as requested.

Additional Editor Comments (if provided):

Please check the manuscript thoroughly for the English language and correct, as required. For your guidance, you can check the reviewers' comments. Thank you for giving us the opportunity to consider your work.

AU: Thank you for your review of our work. We have reviewed the manuscript and checked it carefully for English and grammar. We have also updated the formatting according to the formatting requirements. We apologize for this oversight.

[Note: HTML markup is below. Please do not edit.]

Reviewers' comments:

Reviewer's Responses to Questions

**Comments to the Author**

1. Is the manuscript technically sound, and do the data support the conclusions?

The manuscript must describe a technically sound piece of scientific research with data that supports the conclusions. Experiments must have been conducted rigorously, with appropriate controls, replication, and sample sizes. The conclusions must be drawn appropriately based on the data presented.

Reviewer #1: Yes

Reviewer #2: Yes

2. Has the statistical analysis been performed appropriately and rigorously?

Reviewer #1: Yes

Reviewer #2: I Don't Know

3. Have the authors made all data underlying the findings in their manuscript fully available?

The [PLOS Data policy](http://www.plosone.org/static/policies.action#sharing) requires authors to make all data underlying the findings described in their manuscript fully available without restriction, with rare exception (please refer to the Data Availability Statement in the manuscript PDF file). The data should be provided as part of the manuscript or its supporting information, or deposited to a public repository. For example, in addition to summary statistics, the data points behind means, medians and variance measures should be available. If there are restrictions on publicly sharing data—e.g. participant privacy or use of data from a third party—those must be specified.

Reviewer #1: Yes

Reviewer #2: Yes

4. Is the manuscript presented in an intelligible fashion and written in standard English?

PLOS ONE does not copyedit accepted manuscripts, so the language in submitted articles must be clear, correct, and unambiguous. Any typographical or grammatical errors should be corrected at revision, so please note any specific errors here.

Reviewer #1: Yes

Reviewer #2: Yes

5. Review Comments to the Author

Please use the space provided to explain your answers to the questions above. You may also include additional comments for the author, including concerns about dual publication, research ethics, or publication ethics. (Please upload your review as an attachment if it exceeds 20,000 characters)

Reviewer #1: This is a nicely written paper for the most part, and provides a clear and thorough explanation of the work done. The study is very interesting and tackles a relevant and important area of dairy calf welfare, through using anticipatory behaviour. It is a real shame that, after all the care and attention given to the study design and execution, that the very significant loss of video data has reduced the value and impact of the work. Nevertheless there is enough novel work in here to merit publication, although the outcomes and understanding from the study remain rather weaker than would otherwise have been the case. Overall I have only minor comments regarding some rather wayward use of punctuation in places, and where the clarity of the study could be improved:

AU: We are very appreciative of the Reviewer’s positive feedback on the study. Indeed, we were absolutely gutted by our video system’s massive failure. We have since updated to a new server to avoid this kind of thing happening in the future. Nonetheless, we are grateful that Reviewers have seen merit in the data we were able to salvage. We’ve hopefully thoroughly addressed your comments but welcome any follow-up you may have!

Line 13: End sentence at animals and begin new sentence with However

AU: Changed.

Line 16: Assume this is the data from the 16 pairs of calves for which you did have data rather than the 64 animals you began the study with?

AU: Correct – we chose to just indicate the number of calves for which we had data in the abstract, in the interest of space. We indicate the original number of calves enrolled in the methods section.

Line 23: you variously describe this metric as shifts, changes or transitions in behaviour. Since you also use shifts for 'upshifts' in reward value it would be better to firstly be consistent and secondly probably to stick to either transitions or changes throughout (transitions probably best)

AU: Thank you for this suggestion. We have changed to transitions throughout.

L24: no effect 'on anticipation'

AU: Changed.

L27: end sentence at behaviour, being new sentence with However

AU: Changed.

L41: end sentence at [4,5], begin new sentence with This

AU: Changed.

L45: end sentence at reward, begin new sentence with This

AU: Changed.

L48: end sentence at reward and provide reference; begin new sentence with Thus

AU: Changed and added reference.

L52-55: referencing seems to have changed to a different style? Also in a few other places in the manuscript

AU: Corrected.

L89: end sentence at reward, begin new sentence with However, this...

AU: Changed.

L142: suggest change 'had to' to 'could' since you state at L148 that calves were not forced

AU: Changed.

L167: E-B

AU: Corrected.

L212-217: This is such a shame! At L214-215 can you clarify the animal numbers in terms of B-B, B-E etc?

AU: Indeed – given the time and effort that went into this study, we were grateful to be able to salvage what we did! We have now added animal numbers for each treatment as well.

L238: not clear how latency to leave was defined - add to ethogram?

AU: Thank you. This has been added to the ethogram.

L239-240: clarify this was related to data loss and lack of data not any other decision - these data would have been really beneficial to the study and understanding the responses sadly.

AU: We have added this clarification.

L449: reference style?

AU: Apologies, we have corrected the reference style throughout.

L469-474: Yes, would have really added value to this paper to know what the calves did in the pens to reduce some of the speculation

AU: We agree this would have been ideal but unfortunately was not possible with our data loss.

L487-489: Although the enriched pens were better than the barren pens I would be careful in inferring that the animals were in an unrelieved state of positive welfare, it may be that it was just a bit better. After all they have been separated from their dam, which has been shown to influence judgement bias, and the enriched pens is far from optimal for young calves.

AU: This is a valid point – we have added some consideration around this point to the end of the sentence.

L501: reference style?

AU: corrected

Reviewer #2: Manuscript should be checked carefully for english and grammatical error. Hypothesis is not clear. Introductory section require revision and only relevant information needs to be incorporated. Discussion section needs more rectification.

AU: We have carefully reviewed the manuscript for English and grammar, and have added a clarification regarding our hypothesis. We have cut back some of the introduction to be more focused. With regards to the discussion, while we acknowledge there is a lot of content, we feel it is all relevant to results found, and the story being told; we have therefore left most of it as it was, with the exception of a few changes requested by Reviewer 1. If however Reviewer 2 has some specific suggestions, we are happy to examine and address these.
